# Atypical Presentation of a Splenic Abscess Secondary to Splenic Artery Thrombosis With Isolated Confusion: A Case Report

**DOI:** 10.7759/cureus.114016

**Published:** 2026-08-05

**Authors:** Sarah El Haddad, Paul Boulos, Joe Kadou, Simon Kalisz

**Affiliations:** 1 Emergency Department, Centre Hospitalier Interrégional Edith Cavell, Brussels, BEL

**Keywords:** acute confusion, causes of splenic abscess, emergency medicin, essential thrombocythaemia, jak2 v617f mutation, thromboembolism and splenic infarction

## Abstract

Splenic abscess is a rare but life-threatening condition whose diagnosis is frequently delayed because of its nonspecific presentation. Development of a splenic abscess following splenic infarction secondary to splenic artery thrombosis is exceptional, particularly when revealing an underlying myeloproliferative neoplasm.

An 85-year-old woman presented to the emergency department after three weeks of intermittent confusion without fever, abdominal pain, or gastrointestinal symptoms. While neurological investigations were unrevealing, thoracoabdominal computed tomography unexpectedly demonstrated a large splenic abscess associated with splenic artery thrombosis and bilateral pulmonary emboli. Laboratory tests showed marked systemic inflammation and severe thrombocytosis. Ultrasound-guided percutaneous drainage isolated *Escherichia coli*, and treatment with intravenous antibiotics and therapeutic anticoagulation was initiated. The patient's course was complicated by septic shock requiring intensive care admission but ultimately evolved favorably with prolonged antimicrobial therapy and continuous percutaneous drainage, avoiding splenectomy. Additional investigations identified deep vein thrombosis and confirmed JAK2 V617F-positive essential thrombocythemia as the cause of a systemic prothrombotic state leading to multiple arterial and venous thrombotic events.

This case illustrates how splenic abscess may present solely as delirium in older adults, without localizing abdominal symptoms. Emergency physicians should consider occult intra-abdominal infection in elderly patients presenting with unexplained delirium and inflammatory syndrome. When unusual arterial and venous thromboses coexist, an underlying myeloproliferative disorder should be actively investigated. In selected patients, image-guided percutaneous drainage combined with prolonged antibiotic therapy may provide an effective spleen-preserving alternative to splenectomy.

## Introduction

Splenic abscess is a rare entity, difficult to diagnose, and often associated with high mortality [1]. Usually, it occurs as a consequence of a primary infection such as endocarditis or severe pneumonia. It can also be a complication of a gastrointestinal perforation or an arteriovenous malformation in patients with comorbidities such as diabetes, immunosuppression, hematological malignancies, pancreatic disease, or a history of bariatric surgery [2]. Besides its rarity, diagnosis may be difficult because of the nonspecific clinical presentation, typically characterized by fever and pain [3-5].

We report the case of an 85-year-old woman admitted to the emergency department with subacute confusion. Further investigations revealed a large splenic abscess with ischemic necrosis secondary to splenic artery thrombosis in the setting of previously undiagnosed essential thrombocythemia. The uniqueness of this case lies in its atypical presentation and the nearly incidental nature of the diagnosis.

## Case presentation

**Figure 1:**
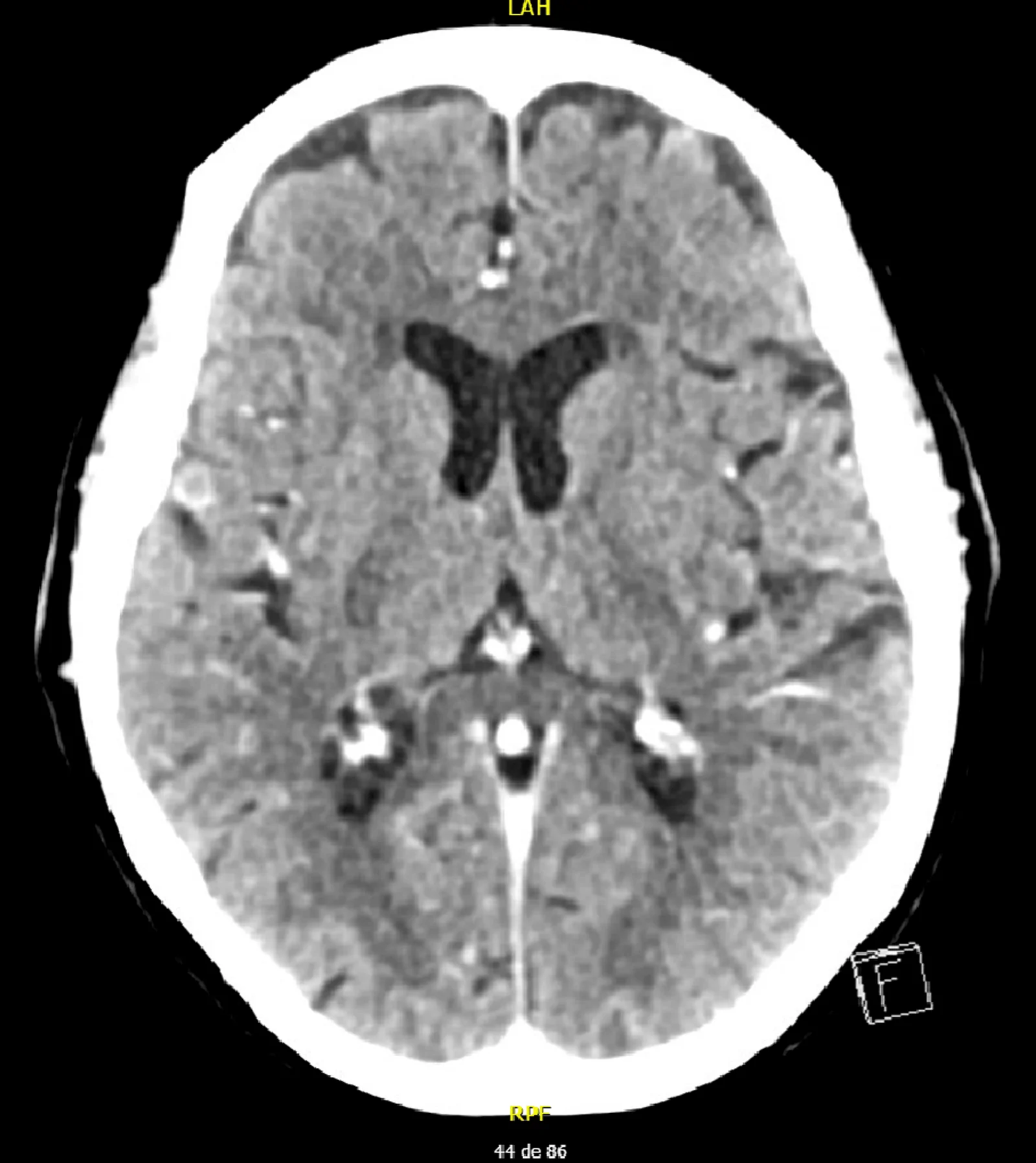
Transversal section of the computed tomography angiography

An 85-year-old woman was referred by her primary care physician for neurological evaluation of intermittent episodes of confusion that had progressed over approximately three weeks. She was priorly known with history of hypertension, hypercholesterolemia, left carotid artery stenosis, and hepatic steatosis, and was taking as usual treatment simvastatin 20 mg, acetylsalicylic acid 80 mg, and perindopril 10 mg. The cerebral computed tomography angiography (CTA) was normal (Figure 1). Further investigations, including blood tests, thoracoabdominal computed tomography (CT), cerebral positron emission tomography-computed tomography (PET-CT), and electroencephalography (EEG), were subsequently requested on an outpatient basis.

**Figure 2:**
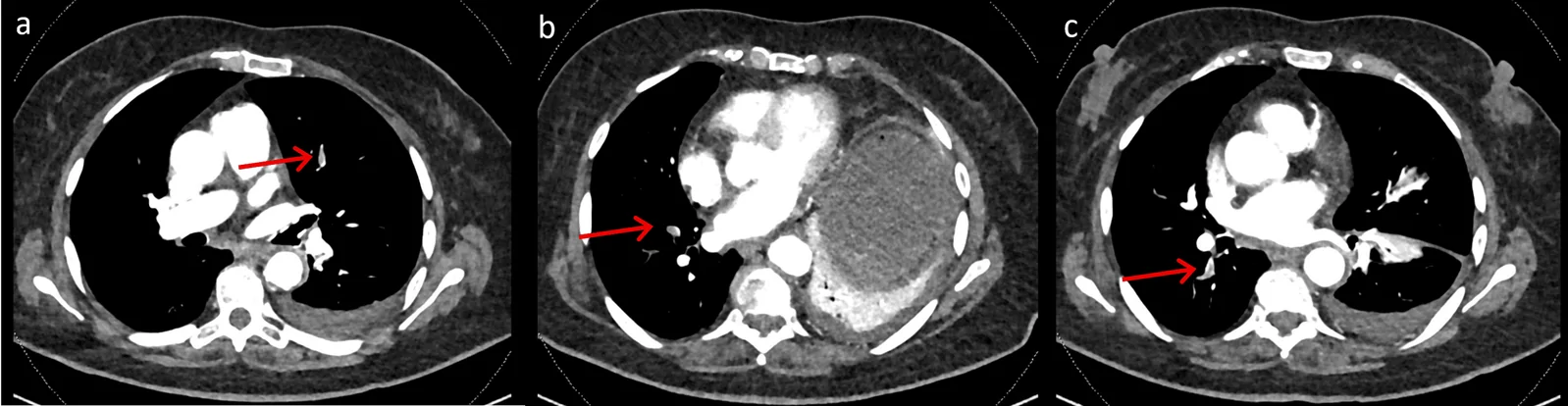
Transversal sections of the thoraco-abdominal CT scan in portal phase; pulmonary embolism indicated by arrows

**Figure 3:**
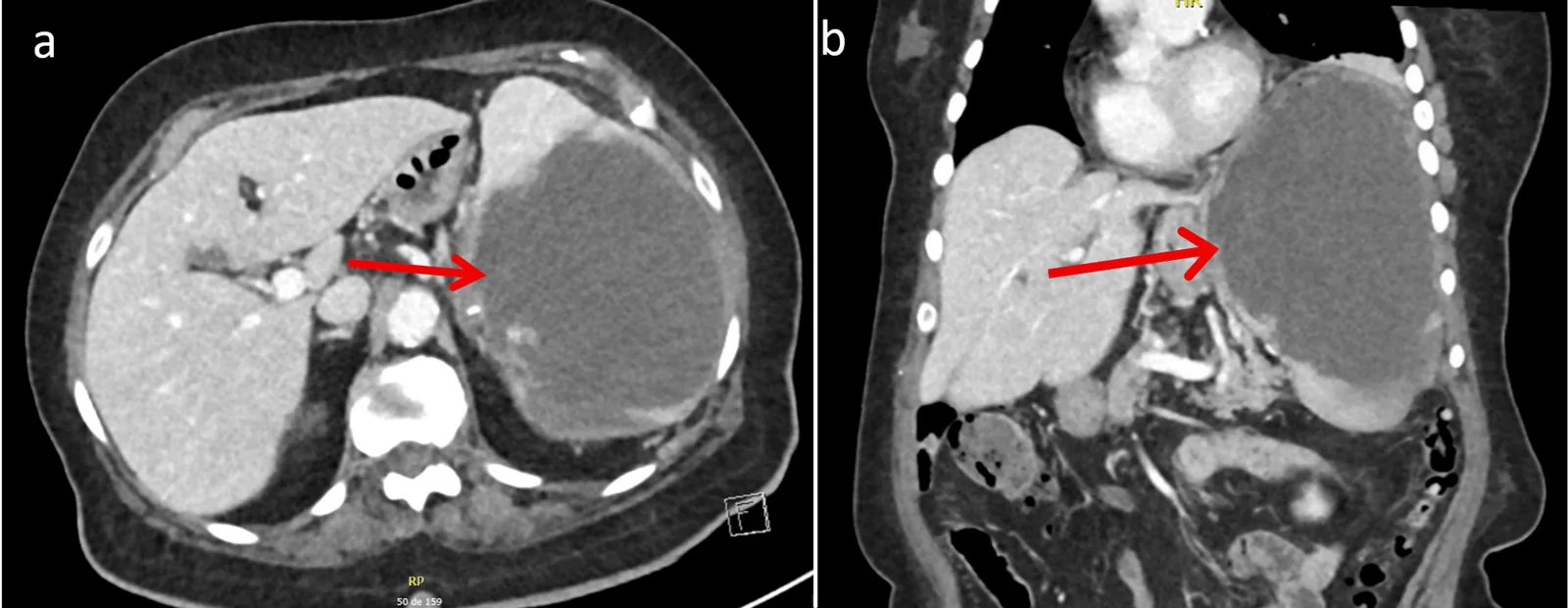
Transversal (a) and coronal (b) sections of the thoraco-abdominal CT scan in portal phase; splenic abscess indicated by arrows

**Figure 4:**
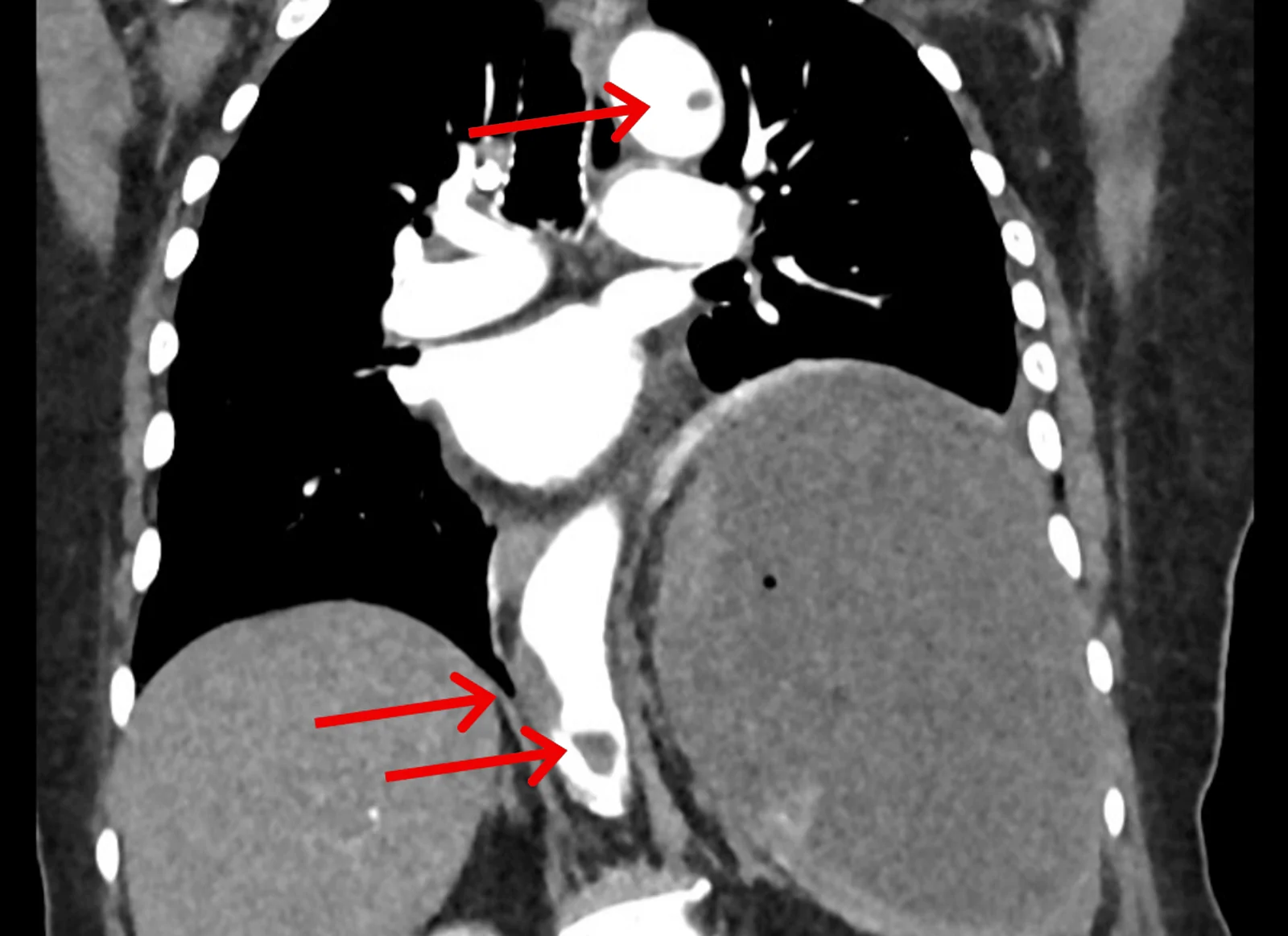
Coronal section of the thoraco-abdominal CT scan in portal phase; multiple atherosclerotic plaques within the aorta indicated by arrows

**Figure 5:**
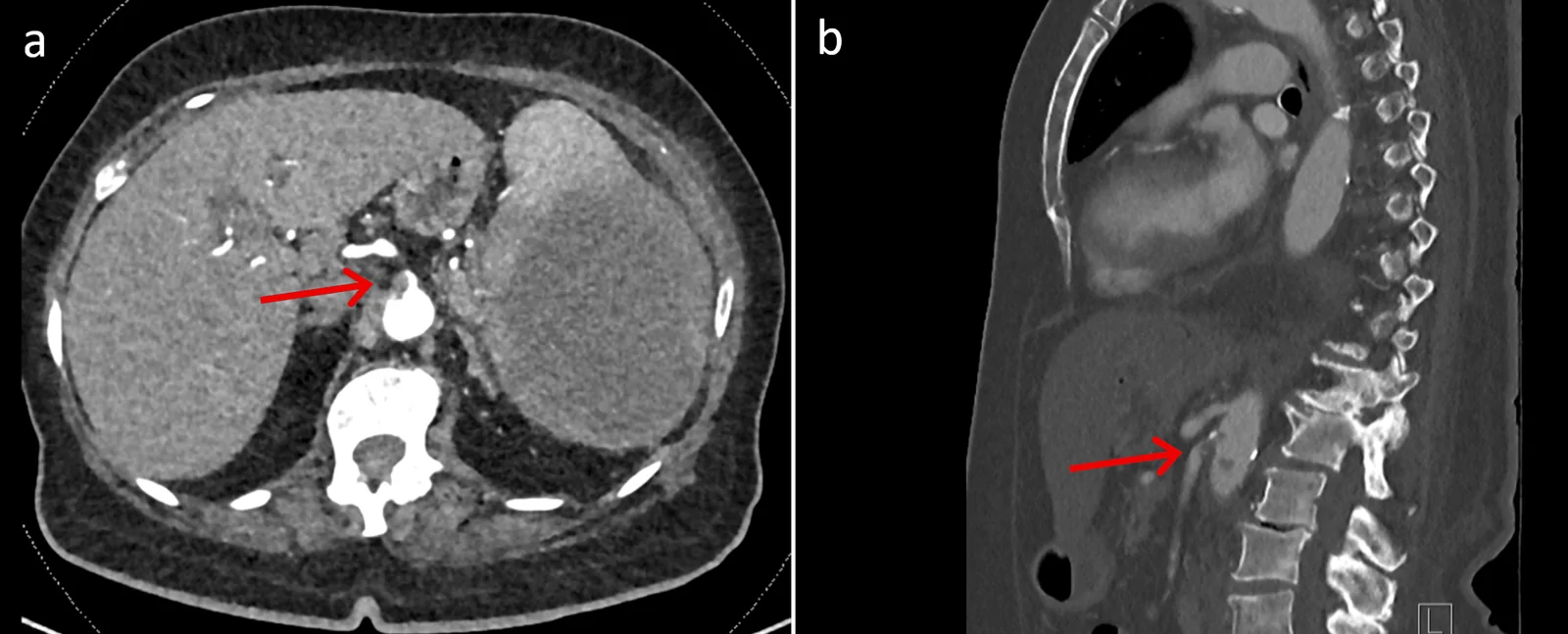
Transversal (a) and sagittal (b) section of the thoraco-abdominal CT scan in portal phase; atherosclerotic plaque at the ostium of the superior mesenteric artery indicated by arrows

Several complementary investigations were scheduled as part of the outpatient diagnostic workup, with the thoracoabdominal CT being performed first according to the available appointment schedule. Although it's not the common approach, it was initially prescribed to investigate an occult infectious source as the cause of her subacute confusion, and unexpectedly revealed bilateral segmental and subsegmental pulmonary emboli (Figure 2), a large splenic lesion containing both fluid and gas suggestive of a splenic abscess (Figure 3), and calcific atheromatosis of the thoracoabdominal aorta with ulcerated soft plaques predominantly at the level of the aortic arch and the aorta at the thoracoabdominal passage (Figure 4), as well as a plaque with estimated 50% ostial stenosis of the superior mesenteric artery (Figure 5). The patient was immediately referred to the emergency department for hospital admission and further evaluation.

At admission, the patient was completely asymptomatic. Clinical examination revealed hypotension (88/65 mmHg) and tachycardia (111 beats/minute). Oxygen level was 93% at room air with a respiratory rate at 18/minute, and her temperature was measured at 37.1°C. She had mild pain upon palpation of the left hypochondrium and hypoventilation at the base of the left lung field. She initially showed no sign of peripherical hypoperfusion. Neurological examination was unremarkable. Laboratory investigations demonstrated a marked inflammatory response, including a C-reactive protein (CRP) level of 153 mg/L (reference value <5 mg/L), hyperleukocytosis of 28.7 × 10³/mm³ (reference range 4.5 × 10³ - 11.0 × 10³/mm³), and high thrombocytosis of 833 × 10³/mm³ (reference range 150 × 10³ - 400 × 10³/mm³) (Table 1).

**Table 1:** Admission blood test results MCV - mean corpuscular volume; MCH - mean corpuscular hemoglobin; MCHC -  mean cell hemoglobin concentration; RDW - red cell distribution; WBC - white blood cells; PT - prothrombin time; INR - International Normalized Ratio; APTT - activated partial thromboplastin time; eGFR - estimated glomerular filtration rate; MDRD - Modification of Diet in Renal Disease; AST - aspartate transferase; ALT - alanine transaminase; GGT - gamma-glutamyl transferase; ALP - alkaline phosphatase; LDH - lactate dehydrogenase; CK - creatine kinase; CRP - C-reactive protein

Analysis	Result	Units	Reference range
Red blood cells	4,114	10^6^/mm³	3.8-5.3
Hemoglobin	11,8	g/dL	11.5-16.1
Hematocrit	35,6	%	35-47
MCV	87	fL	80-100
MCH	28,6	pg	26-35
MCHC	33,1	g/dL	31-36
RDW	15,1		11.5-14.5
Platelets	833	10³/mm³	150-400
WBC	28,7	10³/mm³	4.5-11.0
Neutrophils (absolute)	23,07	×10³/µL	1.8–7.7
Eosinophils (absolute)	0,01	×10³/µL	0–0.45
Basophils (absolute)	0,05	×10³/µL	0–0.20
Monocytes (absolute)	3,58	×10³/µL	0–0.80
Lymphocytes (absolute)	1,49	×10³/µL	1.0–4.8
PT	66	%	>70
INR	1,3		<1.5
APTT	26,6	s	25.1-37
Fibrinogen	571	mg/dL	200-393
Sodium	133	mmol/L	136-145
Potassium	4,8	mmol/L	3.5-5.1
Chloride	99	mmol/L	98-107
Bicarbonate	20	mmol/L	22-29
Calcium	2,11	mmol/L	2.20-2.55
Phosphate	0,98	mmol/L	0.81-1.45
Magnesium	0,77	mmol/L	0.66-0.99
Uric acid	4,6	mg/dL	2.4-5.7
Urea	27	mg/dL	21-43
Creatinine	0,71	mg/dL	0.50-0.90
eGFR (MDRD)	78	mL/min/1.73m²	>60
Total bilirubin	0,3	mg/dL	<1.2
Direct bilirubin	<0.2	mg/dL	<0.3
AST	33	U/L	<32
ALT	28	U/L	<33
GGT	88	U/L	<36
ALP	151	U/L	35-104
Lipase	27	U/L	<60
LDH	375	U/L	135-214
CK	50	U/L	<192
Albumin	30	g/L	35-52
CRP	153	mg/L	<5
Glucose	172	mg/dL	74-109

Empirical intravenous antibiotic therapy with ceftriaxone and metronidazole was initiated, along with therapeutic-dose low-molecular-weight heparin. Urgent ultrasound-guided percutaneous drainage of the splenic collection yielded purulent material. Microbiological culture grew multisensitive *Escherichia coli* less than 24 hours later.

**Figure 6:**
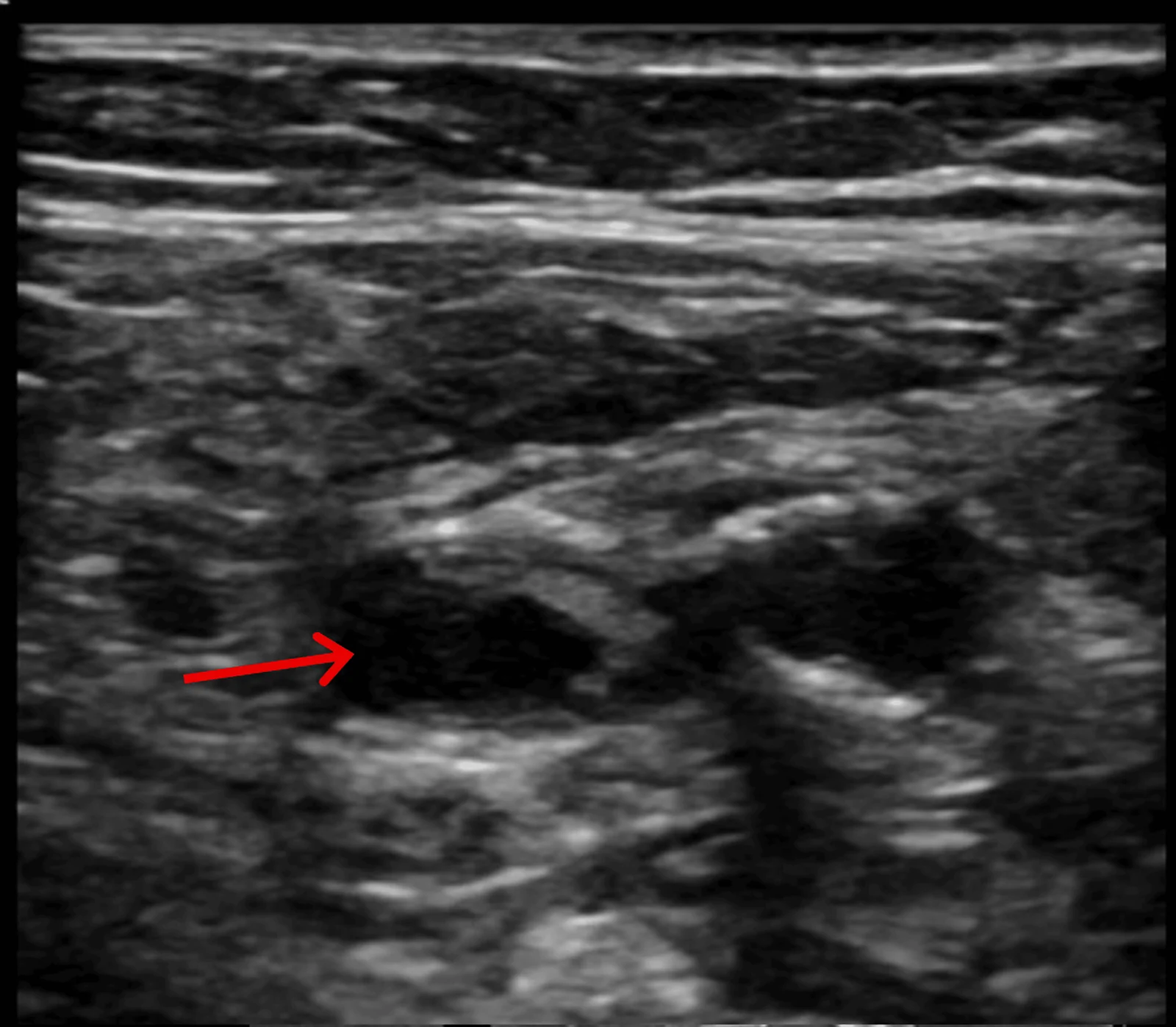
Transversal section of an ultrasonography showing a thrombosis of the left commune femoral vein, indicated by an arrow

**Figure 7:**
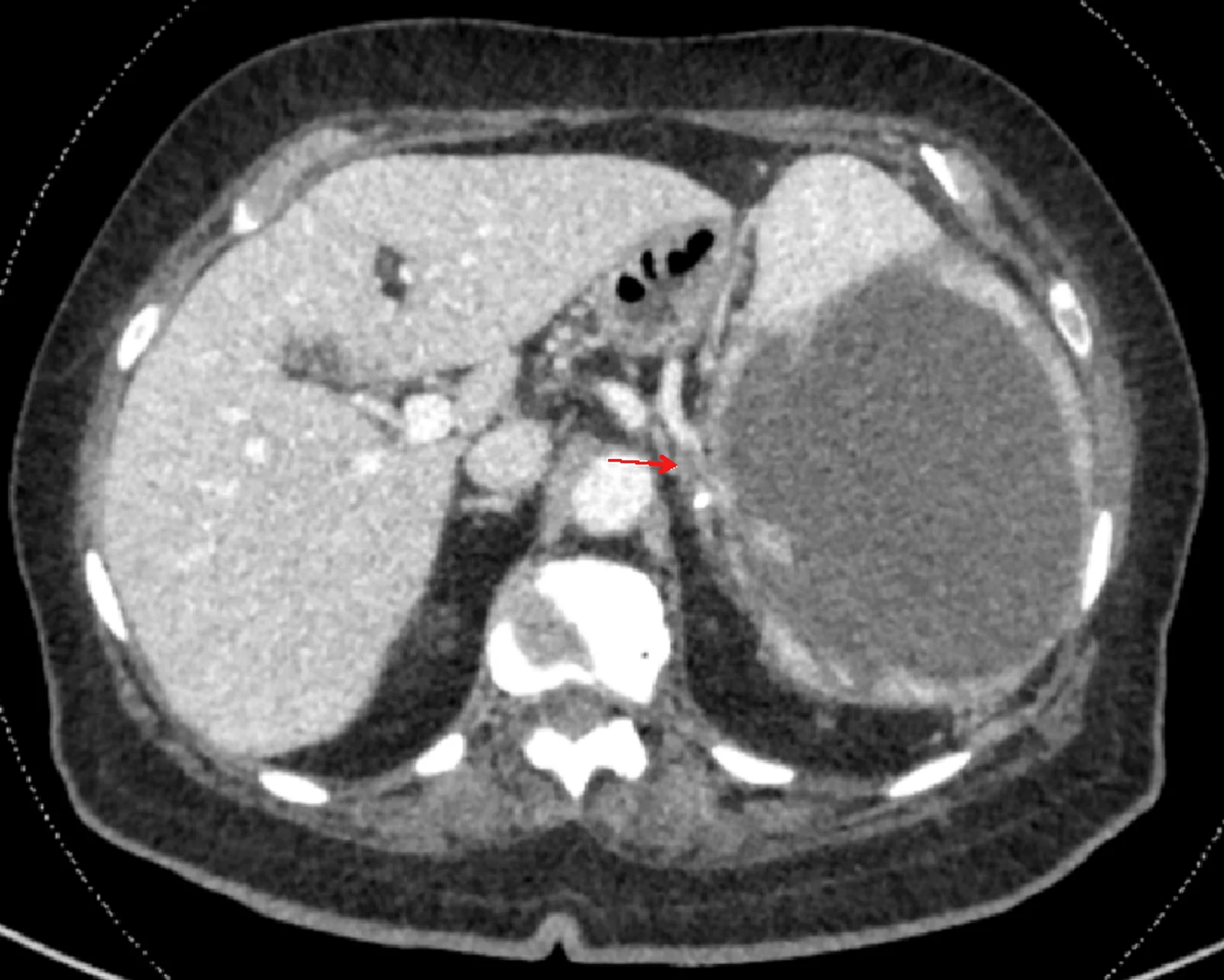
Transversal section of thoraco-abdominal CT scan in portal phase; interruption of the splenic artery indicated by an arrow

**Figure 8:**
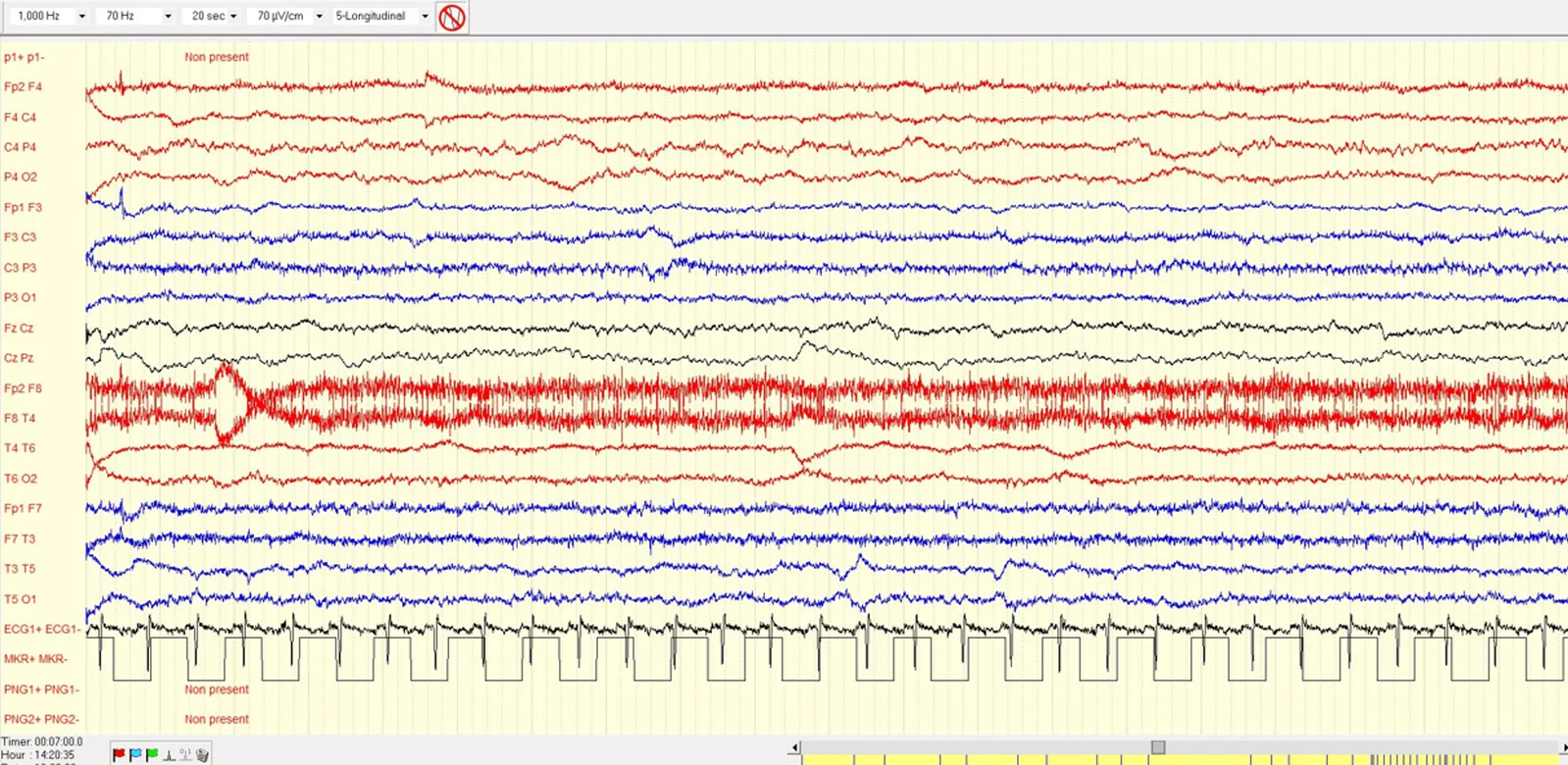
Electroencephalography

**Figure 9:**
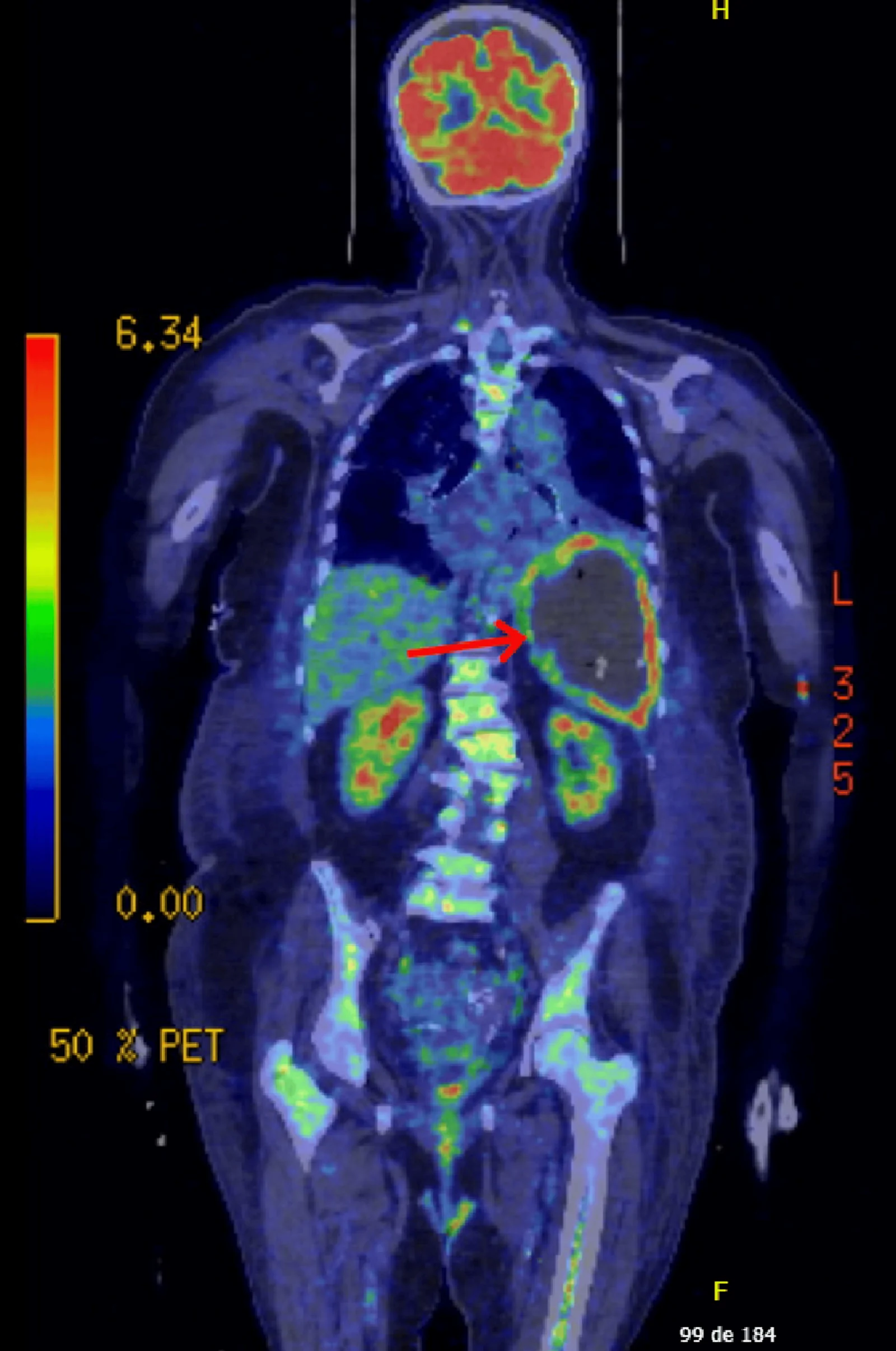
Coronal section of the PET-CT; splenic abscess with hypermetabolic walls indicated by an arrow

Further investigations confirmed multiple thromboembolic events, including deep vein thrombosis of the left femoral vein (Figure 6), bilateral pulmonary emboli without evidence of right ventricular dysfunction, and confirmed the splenic artery thrombosis (Figure 7) due to the calcific atheromatosis previously described. The requested EEG only showed a slowed tracing, consistent with the drug exposure required at the time of the examination (Figure 8). The PET-CT did not indicate any neoplasic lesions, showing only the drained left subphrenic abscess with hypermetabolic walls (Figure 9).

The clinical course was complicated by septic shock and multiorgan failure, necessitating admission to the intensive care unit for six days. Despite the severity of her condition, she improved with prolonged antibiotic therapy and percutaneous splenic drainage, without requiring surgical intervention.

Etiological investigations ultimately revealed an essential thrombocythemia with positive JAK2 V617F mutation, responsible for the thrombophilic state and the occurrence of multiple arterial and venous thrombotic events.

## Discussion

This case highlights several important diagnostic and therapeutic challenges encountered in emergency medicine.

First, the clinical presentation was highly atypical since the patient initially presented with a subacute confusional state in the absence of abdominal pain, fever, or gastrointestinal symptoms. Delirium is a frequent but nonspecific manifestation of severe illness in older adults and may represent the sole presenting feature of serious underlying infection [6-8]. The absence of reported abdominal or infectious symptoms may have contributed to the delayed recognition of the underlying condition. In older patients presenting with unexplained delirium, careful history-taking, including collateral information from family members or caregivers when available, is essential to identify subtle or previously overlooked symptoms. When history and clinical examination do not reveal a clear etiology, further diagnostic evaluation, including appropriate imaging when clinically indicated, may be warranted to identify an occult underlying condition. This case highlights the importance of maintaining a broad differential diagnosis and tailoring the extent of investigations, including imaging, to the individual clinical context.

The pathophysiological sequence observed in this case is unusual. Splenic abscesses are most commonly associated with hematogenous bacterial seeding, bacterial endocarditis, immunosuppression, or contiguous spread from adjacent structures [3-5]. In the present case, splenic artery thrombosis resulted in splenic infarction followed by ischemic necrosis. Secondary infection of the necrotic tissue with Escherichia coli then led to the formation of a splenic abscess. Although this sequence has been previously described, progression from splenic infarction to splenic abscess remains uncommon and highlights the role of thromboembolic events in the development of deep-seated infections [9].

The coexistence of multiple thromboembolic events, including pulmonary embolism, deep vein thrombosis, and splenic artery thrombosis, prompted further investigation for an underlying prothrombotic disorder. Detection of the JAK2 V617F mutation, along with persistent thrombocytosis, established the diagnosis of essential thrombocythemia and confirmed the presence of an acquired thrombophilic state. Myeloproliferative neoplasms are well-recognized causes of both arterial and venous thrombosis and may involve unusual vascular territories [10,11]. In retrospect, splenic infarction was likely the initial manifestation of this prothrombotic condition in our patient.

The optimal management of splenic abscess remains controversial. Historically, splenectomy was considered the standard treatment because of concerns regarding treatment failure and septic complications. However, advances in imaging and interventional radiology have increasingly supported conservative management combining antibiotic therapy with image-guided percutaneous drainage in carefully selected patients [12]. In the present case, continuous percutaneous drainage combined with prolonged antimicrobial therapy resulted in complete clinical recovery, avoiding the need for splenectomy.

Finally, this case highlights the importance of a comprehensive approach in emergency medicine, integrating seemingly unrelated clinical manifestations into a unifying diagnosis [13]. Recognition of a common underlying pathological process was essential for establishing the final diagnosis and guiding appropriate management.

## Conclusions

Splenic abscess secondary to splenic infarction caused by splenic artery thrombosis is a rare but serious cause of sepsis that may present atypically in older patients. Delirium may be the presenting manifestation of a serious underlying condition, even in the absence of localizing symptoms. Careful history-taking, including collateral information when available, combined with a thorough clinical examination, is essential. This case highlights the importance of early imaging and comprehensive diagnostic evaluation in patients with unexplained delirium associated with systemic inflammation. It also illustrates the need to consider acquired thrombophilic disorders, such as JAK2-positive essential thrombocythemia, in patients presenting with multiple thromboembolic events. Although no consensus exists regarding the optimal management of splenic abscess, conservative treatment combining image-guided percutaneous drainage with prolonged antibiotic therapy should be considered in carefully selected patients, as it may represent an effective alternative to splenectomy.
